# Exploring the association between self-efficacy and future utility beliefs in mathematics: A practical tutorial on correspondence analysis

**DOI:** 10.1371/journal.pone.0282696

**Published:** 2023-03-06

**Authors:** Asma Alzahrani, Eric J. Beh, Elizabeth Stojanovski

**Affiliations:** 1 School of Information and Physical Sciences, University of Newcastle, Newcastle, NSW, Australia; 2 National Institute for Applied Statistics Research Australia (NIASRA), University of Wollongong, Wollongong, NSW, Australia; 3 Centre for Multi-Dimensional Data Visualisation (MuViSU), Stellenbosch University, Stellenbosch, South Africa; University of Illinois at Urbana-Champaign, UNITED STATES

## Abstract

It has long been understood that there exists a strong association between a student’s belief in the future utility of mathematics and their self-efficacy in mathematics. This study re-examines this association by studying these variables based on data collected from a sample of 21,444 ninth-grade students who participated in the 2009 High School Longitudinal Study (HSLS09). The nature of the association between future utility beliefs of students in mathematics and self-efficacy of students in mathematics is explored visually using the simple correspondence analysis technique. The main feature that will be utilised from this technique is a two-dimensional graphical display, referred to as a correspondence plot. By studying the HSLS09 data, the first two axes of such a plot summarised nearly 99% of the statistically significant association that exists between a student’s beliefs in the future utility of mathematics and their mathematics self-efficacy. It is shown visually that students who strongly believe in the future importance of studying mathematics also perform strongly in the subject, while those who do not believe that there is any future utility from studying mathematics do not perform well at it. This study, therefore, suggests that mathematics ability is associated with a student’s perception of its future importance.

## Introduction

Mathematics is a key aspect of modern school curriculums and is an academic domain in which student perceptions are particularly important. This is because it has been found that students who demonstrate a higher degree of appreciation for mathematics education are more likely to enrol in additional mathematics courses and hence are more likely to pursue STEM-related careers [1–3]. Mathematics education, therefore, plays an important role in a student’s choice of future career so it is important to understand the factors that influence their academic performance in mathematics which in turn can influence future career choices.

However, behavioural studies conducted on students have linked a student’s attitude and beliefs towards mathematics to how well they are likely to perform or engage in the study of the subject. For example, [4] defines attitudes towards mathematics as “a disposition towards an aspect of mathematics that has been acquired by an individual through his or her beliefs and experiences, but which could be changed”. Indeed, future utility beliefs in mathematics, or how mathematical knowledge pertains to one’s future career or everyday life, also plays a potentially important role in a student’s attitude towards mathematics [5]. Future utility beliefs are defined as beliefs about the perceived personal usefulness of a task [6]. Future utility beliefs in mathematics can thus be regarded as a student’s perception of the importance of mathematics to future job opportunities. Studies have been made of the views of university students about the role of mathematics in their future; see, for example, [7]. According to the results of their survey, some students perceived mathematics as a professional skill that could affect their future studies (33%) and careers (36%). A small proportion of students perceived mathematics as an application of conceptual skills for their future studies (7%) and careers (6%), while some students were uncertain about the role of mathematics for their future studies (7%) and careers (5%). It was recommended that taking subgroups of students enrolled in different degrees at tertiary level could help to clarify reasons for differences in these results [7].

Moreover, self-efficacy in mathematics has also been linked to attitudes towards mathematics. Self-efficacy is a personal quality referring to one’s confidence to perform a certain task competently and describes a learner’s beliefs of their performance at a particular task [8, 9]. It has been claimed that self-efficacy can influence a wide range of behavioural outcomes, including one’s preferences for particular tasks and associated effort expenditure as well as persistence with these tasks in the face of obstacles [8]. Self-efficacy is considered a useful indicator of a student’s attitude as it establishes the relationship between motivation and confidence or beliefs in solving a problem. Academic performance has additionally been found to be closely related to the efficacy of students [8]. The contribution of self-efficacy towards performance in mathematics was illustrated in a study conducted by [10], which found that performance was influenced by a strong foundation in mathematics and a positive attitude towards the subject. Some researchers argue that one’s ability to combine qualities of efficacy determines their performance in mathematics [11–13]. The study of [14] was conducted and focused on determining the effects of engagement and self-efficacy beliefs on mathematics performance. The findings of the study suggest that students with higher efficacy beliefs would perform better in mathematics than those with lower efficacy beliefs. The study also found that students with high mathematics efficacy were more interested and willing to participate in mathematics lessons to accomplish their goals.

Much of the analysis that has been performed in the past to examine the link between future utility beliefs in mathematics and mathematics efficacy have been largely confined to model-based statistical techniques. For example, a study assessing the relationship between mathematics self-efficacy and choice of science-based careers uses a hierarchical regression model [15]. Furthermore, another study used structural equation modelling to examine the nature of the relations between competence beliefs, utility value and achievement goals in mathematics [16]. One may indeed consider a variety of alternative modelling strategies for investigating this relationship. However, in the words of [17, p. 792] “since all models are wrong, the scientist cannot obtain a “correct” one by excessive elaboration”, although [18] conceded later that “all models are wrong, but some are useful”. This is because all modelling strategies rely on an array of varying assumptions about the data or the model itself and largely explore “causation” rather than “association”. This paper will instead explore the nature of the association between these variables using a visual statistical tool that is essentially “assumption-free” and provides the analyst with an intuitive, simple and informative summary of the association. This will be done using a technique called *simple correspondence analysis* (SCA); see [19–22] for technical, practical, computational and historical descriptions of SCA. By using SCA, our analysis of the association between future utility beliefs in mathematics (*Utility*) and mathematics efficacy (*Efficacy*) is conducted using data from the 2009 High School Longitudinal Study (HSLS09) that is summarised in the form of a contingency table. Performing a SCA on this data provides a visual summary of the association between *Utility* and *Efficacy* that enables the analyst to explore how the categories of each variable are linked. Specifically, it highlights how the row and column categories of the contingency table are associated, thereby providing deep insights into the structure of the association between these variables. Such structures can have important implications for the learning and hence the teaching of mathematics in practice. The use of SCA in this context helps to determine the extent of the impact of mathematics efficacy on future utility beliefs in mathematics and can therefore potentially aid to enhance mathematics efficacy if students are made more aware of the usefulness of mathematics in practice, which has the potential to translate to a more positive attitude towards mathematics education. Interestingly, while SCA and its many variants–[23] give an overview of over 30 variants and more have since been identified or developed–is a very useful technique to analyse educational data, it appears to be an underutilised approach in studying a student’s perception of mathematics education. Although, the technique has been employed in other facets of education; see, for example, [24–27].

We highlight that confining our attention to just exploring the nature of the association between two educational variables may provide a limited perspective for some since covariate information concerning, for example, demographic information of the students or school type (public versus private), is not being considered in this study. Additional variables certainly can be taken into account although require multivariate versions of SCA, often referred to as multiple correspondence analysis (MCA) and multi-way correspondence analysis (MWCA); see [28] for a discussion of the key differences between these methods. Since this paper focuses on the application and practical interpretation of SCA for examining the association between *Utility* and *Efficacy* we shall consider the application using MCA or MWCA to the study of more variables for future consideration.

## Methods

### Sample

The High School Longitudinal Study of 2009 (HSLS09) was conducted by the National Center for Education Statistics (NCES) in the United States (US) to monitor the performance of students as they transition from high school to post-secondary studies; all data from this study are available at https://nces.ed.gov/surveys/hsls09/. This website also states the two key roles of the survey. Firstly, the “NCES collected the data necessary to study how incoming 9th graders were led into crucial early math and science courses that affected future coursework necessary for STEM career progress…” and, secondly, for “… developing a better understanding of what factors (eg. previous grades, test scores, parental involvement) relate to who enrols in Algebra I by 9^th^ grade and how this impacted the rest of the high school experience”. The importance of the HSLS09 is still apparent today with an extensive number of peer review publications still appearing over 13 years after it was conducted thereby highlighting the rich source of information it provides. These publications examine a wide range of pedagogical, social, education and policy issues. For example, data from HSLS09 was the focus of studies undertaken by [29–31] which were all published in 2021 while [32–34] appeared in 2022.

The target population comprised of 944 public and private schools, inclusive of public charter schools across all states of the US and the District of Columbia during 2009 [35]. Data collection from ninth-grade students from these schools was undertaken in 2009 and the sampling process was implemented using a two-step stratified sampling scheme involving school recruitment [35]. This scheme involved first dividing the population of schools into strata based on a chosen criterion (such as geographical location), and then acquiring a random sample from each stratum [36]. A total of 21,444 ninth-grade students were selected in the HSLS09 study from these schools.

### Questionnaires

The study was conducted using questionnaires which were administered to the target subjects. Although the student is the primary unit of analysis, HSLS09 also included questionnaire data from school administrators and the students’ parents [35]. A student assessment was also provided on simple algebraic reasoning and was conducted through a computer-assisted telephone interview (CATI) [35]. All stakeholders (students, parents, teachers’ school heads, and lead counsellors) completed the questionnaires through an online platform [35]. This provided information regarding the variables under consideration for the present study which included future utility beliefs in mathematics and mathematics efficacy. An emphasis of the HSLS09 was on the integration into and out of STEM fields in addition to the impacts of such shifts, which may be educational or social in nature.

The information contained in the students’ questionnaire included demographic information and information on school experiences. The questionnaire also covered other aspects such as information concerning high school, student’s career plans, post-secondary plans, and concepts in mathematics, how well students understand them, among other items [35].

### Measures

The two variables that we study in this paper are:

- Future Utility Beliefs in Mathematics (*Utility*): this variable was assessed by asking the following question “Math courses are useful for future careers”. The student was then asked to respond to this question using the following four-point scale: 1 (strongly disagree), 2 (disagree), 3 (agree) to 4 (strongly agree).
- Mathematics Efficacy (*Efficacy*): this variable reflects the personal self-confidence regarding a learner’s ability to excel in certain areas and is considered a measure of academic self-belief [8]. A student’s mathematics efficacy was assessed by asking them to respond to the statement “You are confident that you can do an excellent job on math tests” using the same four-point scale used to assess *Utility*.

In the next section we show how performing a simple correspondence analysis on the contingency table formed from cross-classifying *Utility* and *Efficacy* can be performed. In doing so, we also provide an interpretation of the key features of the analysis and describe the nature of the association between them.

## Simple correspondence analysis (SCA)

### An overview

Table 1 shows the 4 x 4 contingency table obtained by cross-classifying the *Utility* and *Efficacy* variables from the HSLS09. The sample size of Table 1 is 18,795, which is a reduction of the original size of the sample (of 21,444 students) since some students contained missing information for at least one of the variables and so were omitted from our study. The association between *Utility* and *Efficacy* was first analysed using a chi-squared test of independence to determine if a statistically significant association existed between the two variables. The results of this test yield a chi-squared statistic of X^2^ = 2172 which, with a p-value that is less than 0.01, showing that there exists a statistically significant association between the two variables. However, this test does not indicate how the two variables are associated. Therefore, SCA will be applied to Table 1 and can be viewed as a descriptive and exploratory statistical tool that is used to visually analyse the association between two categorical variables [19–22]. When a study consists of multiple categorical variables then multiple and multi-way correspondence analysis can be applied [28]. SCA has practical benefits including its ability to handle data that may not satisfy the necessary data restrictions used in statistical analysis techniques [37]. Furthermore, SCA provides a graphical approach to visualising the rows and columns of a two-way contingency table using points in a low-dimensional space, such that the position of these points reflects the sources of the association that exist in the table.

**Table 1:** Contingency table of *Utility* and *Efficacy*.

Future Utility Beliefs in Mathematics	Mathematics Efficacy				Total
	strongly disagree	disagree	agree	strongly agree	
**strongly disagree**	170	212	239	112	733
**disagree**	163	699	1181	350	2393
**agree**	236	1923	5271	1624	9054
**strongly agree**	102	753	3326	2434	6615
**Total**	671	3587	10017	4520	18795

### Notation and profiles

To briefly describe SCA, consider a two-way (I×J) contingency table, **N**, where the (i, j)’th cell entry is denoted by n_ij_ for i = 1, 2,⋯,I and j = 1, 2,⋯,J. Let p_ij_ = n_ij_/n where n is the sample size so that $\sum_{i=1}^{I}\sum_{j=1}^{J}p_{ij}$ = 1. In terms of the two variables that are being examined for this study, I = 4 represent the four *Utility* categories and J = 4 represent the four *Efficacy* categories; these categories are “strongly disagree”, “disagree”, “agree” and “strongly agree” for both variables. SCA is performed on a two-way contingency table by comparing the relative distribution of counts of the rows and columns in terms of their profiles. The row profiles represent the distribution of the relative joint frequencies for each category of the *Utility* variable and, for Table 1, these are summarised in rows of Table 2. Similarly, the column profiles reflect the distribution of the relative joint frequencies of the *Efficacy* categories; these are summarised in the columns of Table 3. To examine the strength of the association between the row and column categories, we can assess those elements of the row or column profiles that are equivalent to their expected value if it assumed that the *Utility* and *Efficacy* variables are independent. For example, in this situation we would like to determine those elements of the row profiles that are equivalent to the column margins and to see whether the elements of the column profiles are equivalent to the row margins. Any deviation from these equivalencies means that there is an association between our *Utility* and *Efficacy*, and this can be visualised using SCA, as will be described shortly.

**Table 2:** Row profiles; for the variable *Utility*.

Future Utility Beliefs in Mathematics	Row Profiles				Total
	Mathematics Efficacy				
	strongly disagree	disagree	agree	strongly agree	
**strongly disagree**	0.231	0.289	0.326	0.152	1
**Disagree**	0.068	0.292	0.493	0.146	1
**agree**	0.026	0.212	0.582	0.179	1
**strongly agree**	0.015	0.113	0.502	0.367	1

**Table 3:** Column profiles; for the variable *Efficacy*.

Future Utility Beliefs in Mathematics	Column Profiles			
	Mathematics Efficacy			
	strongly disagree	disagree	agree	strongly agree
**strongly disagree**	0.253	0.059	0.023	0.024
**disagree**	0.242	0.194	0.117	0.077
**agree**	0.351	0.536	0.526	0.359
**strongly agree**	0.152	0.209	0.332	0.538
**Total**	1	1	1	1

### Investigating the association

An alternative, but equivalent approach, is to determine those cell proportions that deviate from the model of complete independence, p_ij_ = p_i_. p_.j_ for all i, j = 1, 2, 3, 4. Complete independence will rarely be satisfied, and so a multiplicative measure of departure from this model can be considered. One such model is to consider p_ij_ = α_ij_ p_i_. p_.j_ where α_ij_ = p_ij_/(p_i._ p_.j_), which is referred to as the *Pearson ratio* of the (i, j)’th cell of the contingency table [19–21].

If there is complete independence between the *Utility* and *Efficacy* variables, then all the Pearson ratios of Table 1 will be equal to 1 (α_ij_ = 1 for i = 1,…,4 and j = 1,…,4). Otherwise, the ratios will differ from one and such values reflect the departures from independence. The chi-squared statistic can also be calculated in terms of these ratios such that

$$X^{2}=n\sum_{i=1}^{I}\sum_{j=1}^{j}p_{i.}p_{.j}({\alpha_{ij}-1)}^{2}.$$

Therefore, a zero chi-squared statistic, which supports the hypothesis of complete independence, will be achieved when all α_ij_ = 1. Any deviation from 1 indicates that a *Utility/Efficacy* pair of categories helps to contribute to the association that is present in Table 1. For example,

$$\alpha_{11}=\frac{p_{11}}{p_{1.}p_{.1}}=\frac{\left(170/18795\right)}{\left(733/18795)\times (671/18795\right)}=6.5$$

which is a substantial departure from 1. Therefore, the “strongly disagree” category of both variables helps to contribute to the association between *Utility* and *Efficacy*–this association will be verified visually using SCA to analyse Table 1. If the sample size, n, increases, then so too does the magnitude of the Pearson chi-squared statistic. This can often hinder tests of association in contingency tables because it will lead to statistically significant associations irrespective of the association structure between the variables. To negate the impact of the sample size, a SCA of Table 1 will assess the strength of the association between the *Utility* and *Efficacy* variables by considering the total inertia X^2^/n.

### Visualising the association

The most distinctive feature of SCA is the visual summary that it provides of the association between the categorical variables. To obtain a visual summary of the association between the *Utility* and *Efficacy* variables of Table 1, a generalised singular value decomposition (SVD) is applied to the matrix of Pearson ratio’s so that:

$$\alpha_{ij}-1=\sum_{m=1}^{M}a_{im}\lambda_{m}b_{jm},$$

where M = min(I, J)−1 = 3 is the maximum number of axes needed to visualise the association between the *Utility* and *Efficacy* variables. Here

- The vector $a_{m}={\left(a_{1m},a_{2m},\ldots \ldots ,a_{Im}\right)}^{T}$ is the m’th left (row) singular vector and is associated with the I = 4 row categories of the *Utility* variable. They contain the scores that help identify, along each of the M axes, those row categories that have a similar, and different, profile and are orthogonal with respect **to** the row relative marginal frequencies, p_i._.
- The vector $b_{m}={\left(b_{1m},b_{2m},\ldots \ldots b_{Jm}\right)}^{T}$ is the m’th column singular vector and is associated with the J = 4 column categories of the *Efficacy* variable. They contain the scores that help identify, along each of the M axes, those column categories that have similar, and different, profiles, and are orthogonal with respect **to** the column relative marginal frequencies, p_.j_.
- The elements of the vector ${\left(\lambda_{1},\lambda_{2},\ldots \ldots \lambda_{M}\right)}^{T}$ are the first M singular values and are arranged in descending order. These are the weights associated with each of the M axes so that the first axis always reflects most of the association that exists between the variables, the second axis reflects the next highest amount of the association and so on. It can be shown that the sum-of-squares of these values gives the total inertia so that all the association is captured in the M-dimensional space.

The set of singular vectors, **a**_m_ and **b**_m_, are used to define the coordinates along the m’th axis of a visual summary. However, they do not reflect that association that exists between the variables (they only provide a comparison of the row, and column, profiles). Therefore, the principal coordinates that are used to construct a visual summary of the association between the i’th *Utility* category and the j’th *Efficacy* category along the m’th axis are

$$f_{im}=a_{im}\lambda_{m}$$

$$g_{jm}=b_{jm}\lambda_{m},$$

respectively. For such a plot, the first (horizontal) axis is referred to as the first principal axis, while the second (vertical) axis is called the second principal axis. In addition, for Table 1, we can also determine 100(1–α)% confidence regions to identify those categories that contribute to the association structure and those that do not [38]. If the axes of a two-dimensional correspondence plot are similarly weighted so that λ_1_ = λ_2_, then the confidence regions are (roughly) circular in shape. However, if λ_1_≫λ_2_, as is generally the case, then the confidence regions will be elliptically shaped. One can interpret these regions so that if they overlap the origin then their categories do not make a statistically significant contribution to the association. If a region does not overlap the origin, then its category does make a statistically significant contribution to the association; see [20, Chapter 8; 38] and the references mentioned therein for more details on their derivation and interpretation.

## Results

### Interpretation of the correspondence plot

**Fig 1:**
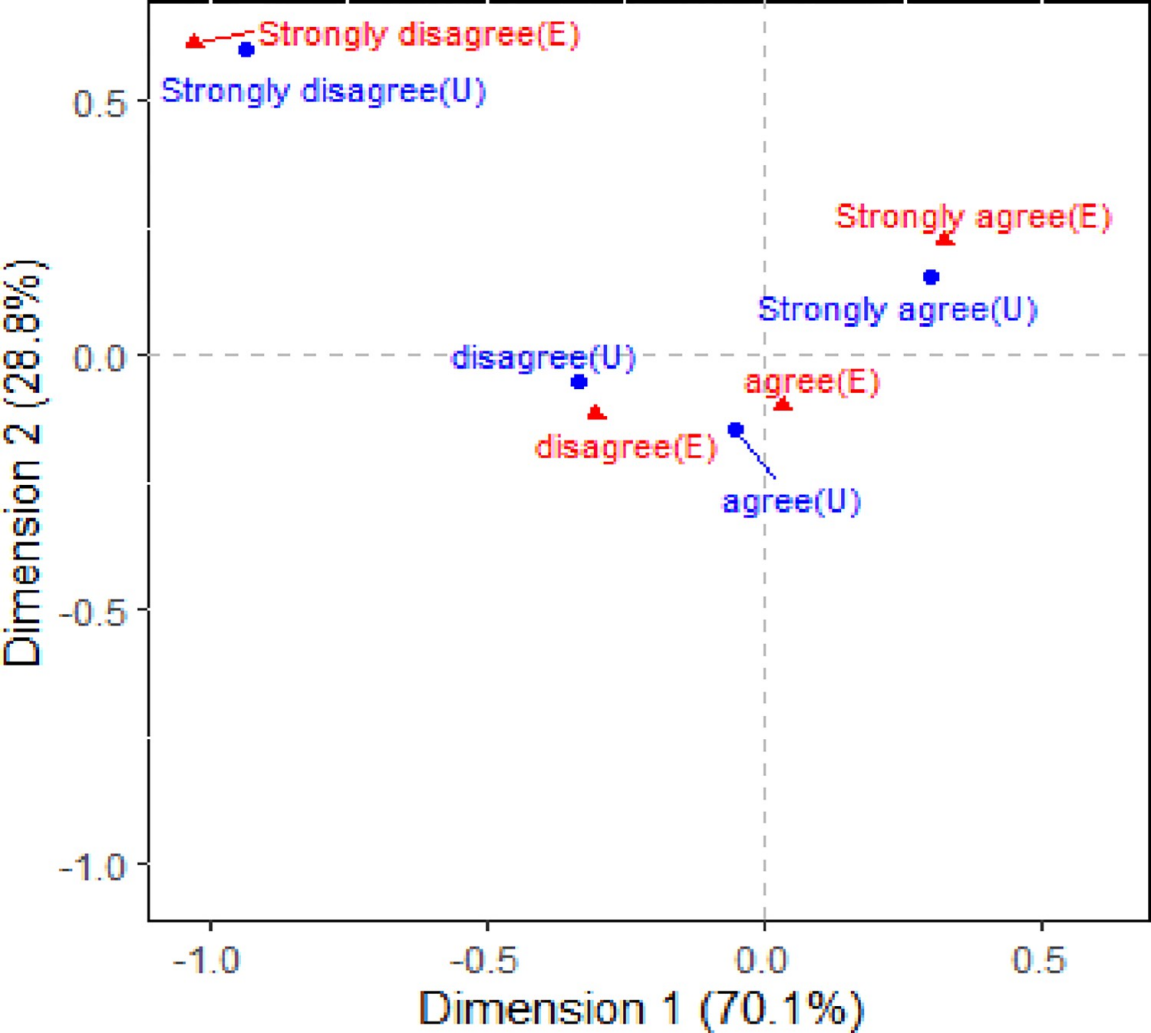
Two-dimensional correspondence plot of Table 1(*Utility* (U) and *Efficacy* (E)).

Recall that there exists a statistically significant association between the *Utility* and *Efficacy* variables based on the data in Table 1 (p-value < 0.01) and this motivates our use of SCA to visually explore the structure of this association. An optimal visual summary of the association consists of M = 3 axes and the first two of them are depicted in the correspondence plot of Fig 1. This figure plots onto the same two-dimensional space the coordinates (f_i1_, f_i2_) for the i’th *Utility* category and (g_j1_, g_j2_) for the j’th *Efficacy* category. The quality of this plot can be assessed by determining the percentage contribution that each axis makes to the total inertia which is X^2^/n = 2172/18795 = 0.12. Table 4 summarises these percentages for the three-dimensional space and shows that the first axis visually accounts for about 70% of the association (λ_1_ = 0.2845) while the second axis accounts for 29% of this association (λ_2_ = 0.1824). These percentage values can be found by determining $100\times n\lambda_{m}^{2}/X^{2}$ for m = 1, 2 and 3. Therefore, Fig 1 visually summarises approximately 99% of the association that exists between the *Utility* and *Efficacy* variables of Table 1. This plot is thus considered to be of excellent quality and is thus very helpful in explaining how the two variables are associated with each other.

**Table 4:** Inertia decomposition of *Utility* and *Efficacy*.

Axis	λ_m_	% of total inertia	Cumulative percent of total inertia
1	0.2845	70.0540	70.0540
2	0.1824	28.7943	98.8483
3	0.0365	1.1517	100.000

We can gain a deeper understanding about the nature of the association between *Utility* and *Efficacy* by observing the relative distance of the points from the origin and from each other. Here, the origin coincides with the position of all the points if there is complete independence between our variables. Therefore, the further a point lies from the origin, the stronger their contribution is to the association. However, it is important to point out that the physical distance between a row point and a column point cannot be quantified, but distances between two row (and column) points can be. So too the distance of a row (and column) point from the origin. With these features in mind, Fig 1 shows that the categories of “strongly disagree” for the *Utility* and *Efficacy* variables are relatively a long way from the origin. This suggests that these categories are the most dominant contributors to the statistically significant association that exists between the two variables. This is also shown by observing the row profile of the “strongly disagree” category in Table 2. From Table 2, the “strongly disagree” category has a very different profile compared to the other *Utility* categories, particularly for the “strongly disagree” and “agree” categories. In fact, we can see that the “agree” category does not consist of profile values that are neither large, nor small, when compared with the values of the other profiles. Rather, the “agree” category of the *Utility* variable is deemed to be an “average” profile. Thus, it’s impact on the association is not considered to be as dominant and for this reason we can see that it is positioned close to the origin of Fig 1.

The column (*Efficacy*) profiles summarised in Table 3 show that there is some variation in their values suggesting that the categories of *Efficacy* contribute to the statistically significant association it has with *Utility* in different ways. In particular, those who “strongly disagree” in the efficacy of mathematics are also far more likely to “strongly disagree” in the future utility of the subject than any other *Utility* category. Hence, this category lies far from the origin in Fig 1 and near the “strongly disagree” category of the *Efficacy* category–recall that the Pearson ratio α_11_ = 6.5 and this verifies the strong association that exists between these two categories that is depicted in Fig 1. Similarly, more than half (53.8%) of those who “strongly agree” in the efficacy of mathematics also “strongly agree” in its future utility, and one can see that these two categories are located close to each other in Fig 1.

An interesting feature of the association between *Utility* and *Efficacy* that is revealed by observing the configuration of points in Fig 1 is that there is a near perfect alignment of the four categories of the variables. That is, students who “strongly agree” that mathematics courses are important for their future career (*Utility*) also “strongly agree” that they are confident that they can perform well in tests on the subject (*Efficacy*). Similarly, those who “disagree” that mathematics is important for their future career also “disagree” that they are confident in performing well under exam conditions. There is also a strong link between those who “agree” with the *Utility* and *Efficacy* questions, which is apparent by noting their close proximity to each other in Fig 1. However, since both points lie close to the origin, this strong link is not a dominant contributor to the association that exists between the two variables. Note we have highlighted above that there is a strong, and dominant, association between those students who “strongly disagree” with the questions asked that monitor their utility and efficacy towards mathematics.

### Confidence regions

**Fig 2:**
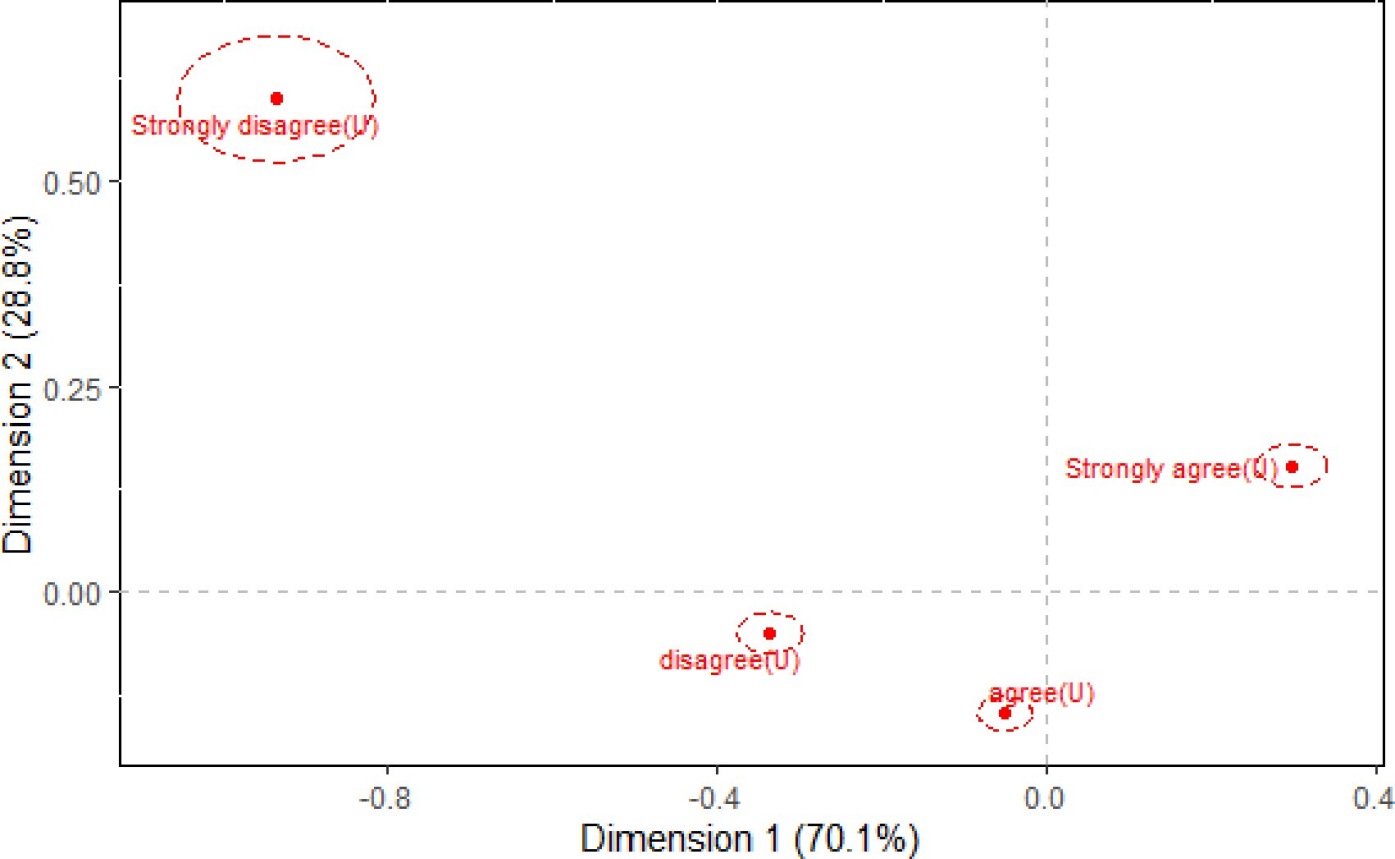
95% Confidence ellipses for the *Utility* (U) variable of Table 1.

**Fig 3:**
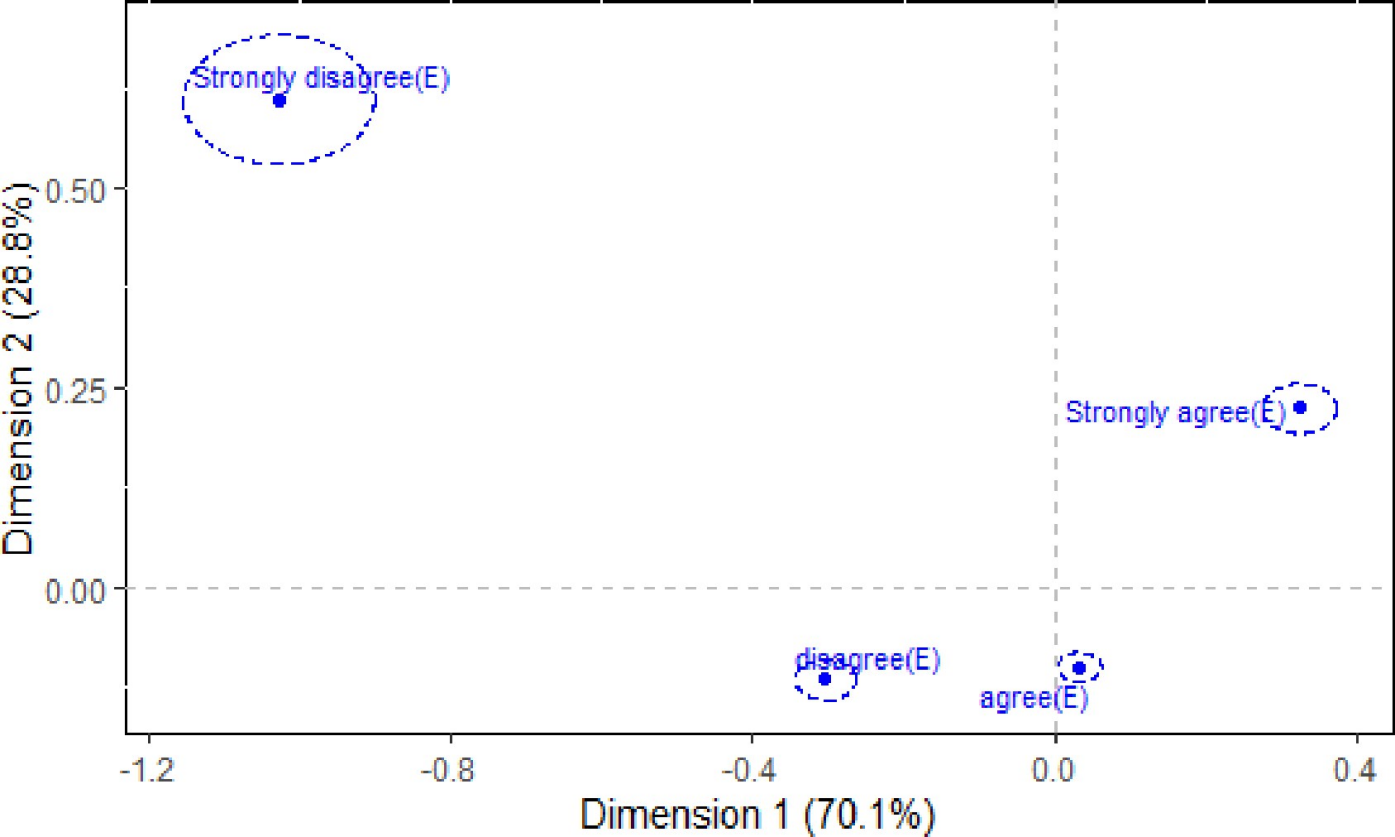
95% Confidence ellipses for the *Efficacy* (E) variable of Table 1.

The dominance, or lack thereof, of a category’s contribution to the association that exists between the *Utility* and *Efficacy* variables can be formally determined using confidence regions. Figs 2 and 3 provide the correspondence plot of Fig 1 with the 95% confidence regions superimposed for each *Utility* and *Efficacy* category, respectively. Based on the three singular values obtained from the study, the first axis reflects more of the association than the second axis, while the second axis reflects more of the association than the third axis (see Table 3); this is due to their property λ_1_>λ_2_>λ_3_. Therefore, since λ_1_>λ_2_, the confidence regions are ellipses where the major axis is parallel to the first (horizontal) axis.

Figs 2 and 3 show that the confidence regions for each of the *Utility* and *Efficacy* categories do not overlap the origin. Therefore, while some categories play a dominant (and not-so dominant) role in the association structure between the *Utility* and *Efficacy* variables, they all have a statistically significant role to play in defining the association. The importance of this finding is that all responses to the two questions are important and help shape our understanding of how the future utility of mathematics and its efficacy are linked.

## Discussion

This study has demonstrated how SCA can be used to identify the strength of the association between future utility beliefs in mathematics and mathematics efficacy using data from the 2009 High School Longitudinal Study (HSLS09). SCA provides a unique graphical understanding of the association that a chi-squared test cannot reveal. One could consider determining the correlation between the *Utility* and *Efficacy* variables, but such a measure is generally limited since it assumes that a linear relationship exists between the variables (thereby masking any non-linear sources of association that might exist) and will not reveal how the responses to the *Utility* question are related to the responses of the *Efficacy* question; SCA provides a visual solution to the problems inherent with both of these more popular numerical statistical tools.

As we have shown in the correspondence plot, the higher the opinion that a student has towards mathematics efficacy, the more they agreed to the importance of mathematics in future utility beliefs. In contrast, students with low efficacy were more likely to disagree with the importance of mathematics in future utility. It can therefore be deduced that, generally, the better a student performs in mathematics, the more likely they are to take up careers related to mathematics. These conclusions are consistent with the findings of [14] who also found that students with high self-efficacy beliefs in mathematics had a higher engagement in the subject, implying an association between beliefs in mathematics and efficacy for mathematics. This study can potentially assist educators to determine the preferred teaching methods and strategies to adopt in the learning environment to improve their students’ understanding of the importance of mathematics in future careers. Such strategies can then assist students in gaining a deeper insight on the importance of mathematics in their future careers. This can also potentially encourage students to want to perform better in mathematics and therefore to increase their mathematics efficacy.

A word of warning, however, is needed when interpreting plots like that presented by Fig 1. Pearson’s statistic implies that the variables *Utility* and *Efficacy* are symmetrically associated. That is, both variables are considered independent variables and none are treated as a response variable to any other variable studied. Therefore, it is important to keep in mind that Fig 1 is a visual representation of *association* and not of *causation*. So, it is not possible to imply, for example, that those who strongly disagree in the efficacy of mathematics will strongly disagree in its utility. If one does wish to investigate causation in correspondence analysis then they can consider the variant called non-symmetrical correspondence analysis [39, 40] which has at its numerical heart the Goodman-Kruskal tau index [41] instead of Pearson’s famous chi-squared statistic.

While attention in this paper has been confined to examining the association between two variables (*Utility* and *Efficacy*) there are clearly other factors that impact upon them both, including gender, socio-economic background and parental/family influences. For such examinations, multiple and/or multi-way correspondence analysis [20–22, 28] can be adapted to study educational data for future research. Doing so will provide an understanding of the role and influences of other factors on the association between the future utility of mathematics and self-assessed mathematics efficacy in the context of education research.

If we now turn our attention to briefly addressing the comment made by the authors in the introduction where it was stated that SCA (like many of its variants) is largely “assumption-free”. This is indeed the case and a major reason for the appeal of using CA to explore association structures in the data; however, the word *largely* cannot be ignored. There are subtle assumptions that may be deemed not too concerning but should nonetheless be considered, despite most research failing to consider its impact. One such assumption concerns the Pearson chi-squared statistic which assumes that the cell counts of a contingency table are Poisson random variables. This implies that, for a given cell count, its expectation and variance are identical. However, it has been discussed by, for example, [42, 43, pp. 81–82] that this assumption is rarely satisfied and that contingency tables are prone to over-dispersion. Remedies for dealing with over-dispersion in correspondence analysis are very limited, but there are strategies that have been recently considered for overcoming them (either directly, or indirectly). For example, [44] propose five strategies for dealing with overdispersion in correspondence analysis and these were further investigated in [45, Chapter 11]. One may also deal with overdispersion by stabilising the variance. Such a strategy has been discussed by [46–49] and involves using the Freeman-Tukey statistic [50] as an alternative to Pearson’s statistic.

An examination of the nature of the *Utility* and *Efficacy* variables, like many of the variables from HSLS09, are ordinal. Therefore, variants of SCA can be considered that incorporate this structure. For example, [51] provides a method of SCA that utilises orthogonal polynomials as a substitute for left and right singular values and generalised correlations [52] instead of singular values. Generalisations of this method to multiple ordinal variables were examined by [53]. The computation of the ordered approach to SCA, and further adaptations to this method, have been described by [54]. These techniques require more technical rigour than can be discussed in this paper and so examining whether overdispersion is present in Table 1 and, if so, how to accommodate for it will be left for future consideration.
